# George Christopher Tayler

**Published:** 1912-12

**Authors:** 


### Obituary Notices.

---

### GEORGE CHRISTOPHER TAYLER, M.D. Lond., M.R.C.S. Eng., L.R.C.P. Lond.

WITH much regret we record the death of Dr. George Tayler, which took place on November 23rd, at Lovemead House, Trowbridge. On Wednesday he was at work, apparently in good health, on Thursday he was taken ill, and rapidly becoming worse, died early on Saturday morning.

Born in 1845, the son of Dr. Christopher Tayler, of Trowbridge, he studied at Bart.'s, and became a member of the Royal College of Surgeons and a Licentiate of the Royal College of Physicians in 1866. In the same year he graduated as Bachelor of Medicine, London University. He at once commenced practice in his native town, his brother, Dr. H. P. Tayler, of Bradford-on-Avon, joining him in practice some years later. More recently his sons, Christopher and Ernest, joined the partnership. In 1871 he took his degree of Doctor of Medicine.

For many years he was Medical Officer of Health to the town, and a district medical officer to the Trowbridge and Melksham Union, and till the day of his death Medical Officer for the third district of the Westbury and Whorwellsdown Union. He was from its earliest days a member of the staff of the local Cottage Hospital. When in 1903 the Trowbridge Division of the British Medical Association was formed, he was properly and naturally elected its first Chairman,and for the Session 1907–8 he was President of the Bath and Bristol Branch.

An active and enthusiastic volunteer, he retired not many years ago with the rank of Hon. Surgeon Lieut.-Colonel. A much-valued member of the congregation, he was churchwarden at St. Thomas's Church, where on November 27th the first part of his burial service took place, in the presence of a large congregation of mourners from the town and neighbourhood, including many members of the medical profession in Wiltshire.

Dr. Tayler was one of those human, well-learned and common-sense practitioners whom the profession and the public can alike ill spare, one whom it was a privilege to know; always genial, he had that simple, unassuming dignity that so well befits a doctor. Thoughtful, upright, untiring, unselfish, he was honoured and beloved by all who knew him. It has been said that after death a man's bad deeds are writ in brass, his kindnesses in water. All who knew him will realise that in the record of George Christopher Tayler there will be no letters of brass, and those who were present at his burial must have felt that the tears of a multitude of friends and patients were burning into their hearts the memory of the untold k,indnesses of a lifetime, and engraving there a record that will not tarnish, whose influence will not perish.

